# A rapid and effective nonsurgical artificial insemination protocol using the NSET™ device for sperm transfer in mice without anesthesia

**DOI:** 10.1007/s11248-015-9887-3

**Published:** 2015-06-12

**Authors:** Barbara J. Stone, Kendra H. Steele, Angelika Fath-Goodin

**Affiliations:** ParaTechs Corporation, 1122 Oak Hill Drive, Lexington, KY 40505 USA

**Keywords:** Artificial insemination, Nonsurgical, NSET device, In vitro fertilization, Sperm, Anesthesia

## Abstract

Artificial insemination (AI) is an assisted reproductive technique that is implemented successfully in humans as a fertility treatment, performed extensively for commercial breeding of livestock, and is also successful in laboratory rodents. AI in the mouse may be especially useful for breeding of transgenic or mutant mice with fertility problems, expansion of mouse colonies, and as an alternative to in vitro fertilization. Nonsurgical AI techniques for the mouse have been described previously but are not often implemented due to technical difficulties. Here we compare various protocols for preparation of CD1 recipients prior to AI for naïve (in estrus), ovulation-induced, and superovulated females. Timing of hormone administration relative to sperm delivery is also compared. An improved protocol for nonsurgical AI in mice is described, which incorporates a convenient hormone administration schedule for female recipients and rapid, non-stressful sperm transfer without the need for anesthesia or analgesia.

## Introduction

Artificial insemination (AI) is used to assist in reproduction by directly delivering motile sperm to the female reproductive tract. Artificial insemination in mice can be useful as an alternative to breeding for strains which are not easily bred due to physical or behavioral issues. The techniques of AI and in vitro fertilization (IVF) can both be used to generate viable embryos. Development after AI simply continues in the recipient female, while embryos generated from IVF must be transferred to a pseudopregnant recipient. AI or IVF can be used to generate embryos after cryopreservation of sperm, making them useful for cryorecovery of mouse strains. IVF requires collection of a large number of oocytes, fertilization is performed in vitro, and embryos are then transferred into a suitable recipient host for development. The process can be time consuming, technically challenging, and the resources expensive. AI is a convenient alternative to IVF for some applications. In theory, the AI technique is quite simple; sperm are directly added to the reproductive tract of the recipient female and all further development occurs in vivo. Historically, however, procedures for AI in mice have had variable rates of success, tend to be complicated, and as a result are underutilized.

AI can be performed either surgically or nonsurgically. The surgical procedure has the benefit of using a small volume of sperm injected directly into the oviduct (Nakagata 1992; De Repentigny and Kothary 1996) or bursa (Sato and Kimura 2001; Sato et al. 2002) to produce viable embryos. Surgical AI using previously cryopreserved sperm has also been used to generate live pups (Nakagata 1992). However, this procedure requires surgical training and use of anesthesia and analgesia. The surgical procedure requires opening of the abdominal cavity to reach the female reproductive tract, an invasive procedure requiring pain relief and post-operative supportive care.

The use of various nonsurgical methods has also been previously reported. AI has been performed with varying degrees of success in laboratory mice (*Mus musculus*) to produce implanted fetuses (Dziuk and Runner 1960; Leckie et al. 1973). Dziuk and Runner (1960) describe a transcervical technique using a blunt needle and a glass speculum to transfer sperm to mice, which then receive a daily progesterone administration. Leckie et al. (1973) describe a procedure to transfer sperm transcervically with a blunt needle, with the use of an artificial penis and a vaginal tampon (as an alternative to pairing with a vasectomized male to induce a pseudopregnant state) to increase the efficiency of embryo implantation. More recently, nonsurgical transcervical AI has been performed under general anesthesia for production of embryos in superovulated deer mice (*Peromyscus maniculatus*) (Veres et al. 2012). While techniques for assisted reproduction in *Peromyscus* have generally lagged behind techniques available for *Mus*, attempts are being made to refine methods for embryo recovery and in vitro culture (Veres et al. 2012). To this end, a nonsurgical AI technique introducing sperm into the vagina without anesthesia has also been described for *Peromyscus* for use with females in estrus or with superovulated females (Behringer et al. 2014; Duselis and Vrana 2007). However, data from the nonsurgical AI procedures regarding the production of live pups is not available.

The laboratory mouse community is in need of a simple user-friendly method for AI, one that also creates an ethical standard for treatment to mice. To develop this protocol, we first examined some variable aspects of the previously described surgical and nonsurgical AI techniques available for *Mus*. (1) Hormone administration can be used to induce timed estrus and ovulation to generate oocytes for fertilization. However, superovulation has been shown to have adverse effects on embryonic and fetal development (Beaumont and Smith 1975; Ertzeid and Storeng 1992; Van der Auwera and D’Hooghe 2001; Van der Auwera et al. 1999). (2) Timing of hormone administration relative to sperm delivery needs to be considered. Given a natural estrus situation, ovulation and fertilization is thought to occur around midnight, during the dark cycle (Behringer et al. 2014). Oocytes generated after hormone induced ovulation are most efficiently fertilized from surgical AI 7–12 h after administration of hCG, corresponding to timing of ovulation (Sato et al. 2004). This timing of ovulation and fertilization is rather inconvenient for routine use of AI. (3) Induction of pseudopregnancy in the recipient before (Nakagata 1992; De Repentigny and Kothary 1996; Sato and Kimura 2001; Sato et al. 2002), during (Leckie et al. 1973), or after (this study) insemination is required for fetal development. These three variables have made use of techniques for AI in mice problematic.

While surgical and nonsurgical techniques for AI have been developed for use in mice, adoption of these techniques for routine use has not occurred. Therefore, our goal was to develop a simple and convenient protocol for AI in mice that would result in the birth of healthy pups. To that end, we compared various protocols for preparation of female recipients prior to AI; naïve (in estrus), ovulation induced, and superovulated females. Sperm transfer was performed with the NSET device, typically used for nonsurgical embryo transfer in mice (Green et al. 2009; Bin Ali et al. 2014), because it has been shown to be less stressful to mice than a comparable surgical procedure (Steele et al. 2013). Timing of hormone administration relative to sperm delivery was also compared. As a result, an improved protocol for nonsurgical AI in mice is described. This protocol incorporates a convenient hormone administration schedule for female recipients and a rapid, non-stressful sperm transfer, without the need for anesthesia or analgesia. The protocol can be used to produce large litters of healthy pups.

## Methods

### Animals

All animal research was approved by the ParaTechs Institutional Animal Care and Use Committee and conducted under the standards dictated by the Office of Laboratory Animal Welfare, National Institutes of Health, Public Health Service, United States Department of Health and Human Services. All mice were obtained from Charles River Laboratories (Wilmington, MA, USA). Vasectomized male CD1 mice (>2 months old) were used to induce pseudopregnancy. Female CD1 mice between 2 and 5 months old were used as sperm recipients. Male C3H mice (>2 months old) with proven fertility were used as sperm donors. The vivarium was maintained at 20–22 °C with an average relative humidity of 35–50 % under a 12:12 h light:dark (light from 8 am to 8 pm) cycle. Mice were housed in standardized ventilated microisolation caging (Innovive, San Diego, CA, USA). Mice had access to Teklad Global 19 % protein extruded rodent diet #2919 (Harlan Laboratories, Madison, WI, USA) and bottled sterile water (Innovive) ad libitum.

### Sperm isolation and preparation

Human tubal fluid medium (HTF) (Irvine Scientific, Santa Ana, CA, USA, Cat #90126) was prepared by adding 4 mg/ml bovine serum albumin (BSA) (Sigma-Aldrich, St. Louis, MO, USA, Cat #A3311), and filter sterilizing. The HTF media was equilibrated prior to use for at least 30 min at 37 °C in the presence of 5 % CO_2_. Fresh sperm was collected from the cauda epididymis and vas deferens of C3H males. The sperm was capacitated in 500 µl freshly prepared HTF media under embryo culture grade mineral oil (Sigma-Aldrich, Cat #M8410) at 37 °C in the presence of 5 % CO_2_ for 45 min.

### Hormone administration

Pregnant mare serum gonadotropin (PMSG) and human chorionic gonadotropin (hCG, Cat# AFP8456A) were obtained from the National Hormone and Peptide Program, National Institute of Diabetes and Digestive and Kidney Diseases, (Torrance, CA, USA) and diluted in sterile phosphate buffered saline (PBS) (Butler-Schein, Dublin, OH, USA, Cat #002477) to 1000 IU/ml, and stored at −80 °C until use. Female CD1 mice for this study were either naïve (chosen by outward appearance to be in estrus) or were injected with hormones for induction of estrus or superovulation. For naïve females, estrus cycle indicators were observed as previously described (Behringer et al. 2014). Superovulated females were prepared by intraperitoneal (i.p.) injection with 5 IU PMSG and hCG administered ~47 h apart at various time schedules relative to the vivarium light–dark cycle. To induce a more natural ovulation condition in CD1 females, the hormones were reduced to 1 IU per i.p. injection.

### Artificial insemination

After optimization of the procedure, a total of 40 µl of fresh sperm was transferred to the uterine horns of female CD1 mice. The transfer was performed without the use of anesthesia or analgesia and took <1 min per mouse. Sperm transfer was carried out using a commercially available device originally developed for nonsurgical embryo transfer in mice, the NSET™ device (ParaTechs, Lexington, KY, USA). The NSET device, a tapered Teflon catheter connected to a specially designed hub, was attached to a Rainin P20 pipette (Mettler-Toledo, Columbus, OH, USA). The device was loaded with 20 µl sperm from the edge of the sperm sample, avoiding clumps. The un-anesthetized recipient female was placed on a wire bar cage top and allowed to grasp the surface with its forefeet. The mouse was held at the base of the tail using a thumb and forefinger, and the rear angled slightly upward, while lightly pressing the base of the hind legs with two fingers from the same hand. The holding technique has been described previously for nonsurgical embryo transfer (Green et al. 2009) and a video of the procedure is available (www.paratechs.com/nset/). Either the small or large speculum included with the device was used to hold open the vagina. If the larger diameter speculum was used, the cervix could be visually located with the aid of a direct light source. The NSET tip was inserted through the cervix and into the uterus. Once the NSET hub contacted the speculum, the sperm was expelled by pressing the pipette plunger to the first stop. The procedure was then repeated to deliver a total sperm volume of 40 μl. The speculum was removed, and the mouse was immediately paired with a vasectomized male overnight. No post-procedure monitoring was required.

## Results

Initial attempts to perform AI using the NSET device for sperm transfer followed a previously described protocol for nonsurgical insemination (Nagy et al. 2003) with the exception that sperm transfer occurred at the time of hCG injection (instead of 13.5 h after hCG injection). The females were injected i.p. with 0.5 or 1.0 IU PMSG at 1 pm 2 days prior to sperm transfer and 0.5 or 1.0 IU hCG at noon the day of sperm transfer. The NSET device was used to transfer 15–20 µl sperm just prior to hCG injection. The recipient was immediately housed overnight with a vasectomized male to mate, simulating normal breeding behavior. However, no implantation sites were observed (N = 17) and no pups were delivered (N = 13) following these initial pilot studies.

To determine if fertilization of oocytes could occur with this method, the protocol was modified and the female recipients were injected with 5 IU of each hormone to induce ovulation as before. After sperm transfer and pairing overnight with a vasectomized male, female recipients were euthanized and oviducts were flushed with M2 medium. All AI procedures resulted in viable embryos, with an average of 9.7 blastocysts developed after in vitro culture from an average of 30.3 oocytes per ovulation (N = 20). However, using this same protocol for insemination, only three AI procedures resulted in implanted embryos (N = 21). These results indicated that the sperm transfer was successful in that oocytes were fertilized and viable embryos were being produced. However, implantation of embryos in the uterine horns was inefficient.

Since the oocytes were being fertilized but implantation was restricted, it was thought that the amount of hormones administered for ovulation and/or the timing of administration needed to be altered. In confirmation, the first successful demonstration that the NSET device could be used for AI to produce live pups was performed using an alternate injection schedule (A. Harman, personal communication, Children’s Hospital of Philadelphia, PA, USA). 5 IU PMSG was injected at 8:30 pm 3 days prior to sperm transfer and 5 IU hCG at 8:00 pm 1 day prior to sperm transfer. Sperm volume was increased to 40 µl; transferring this volume required two transcervical sperm deliveries per female. The AI procedure produced a litter of pups in a strain of obese mice (that was unable to copulate). In our hands, this protocol resulted in a pregnancy rate of 36 % (N = 19) (Table 1; Trial 9) in female CD1 mice, with litter sizes ranging from 2 to 9 pups, and an average of 4.5 pups per litter. However, pup mortality was high (57 %) with only 13 of 32 pups surviving to weaning. To increase pregnancy rates and to find a more convenient work schedule, other hormone injection and sperm transfer times were tested using PMSG and hCG at concentrations of 5 IU. No increase in pregnancy rate was achieved after administration of 5 IU of hormones prior to AI, although an increase in litter size (10 pups on average) and an improvement in survival rate (to 100 %) were observed when the injection schedule was begun earlier in the day (Table 1; Trial 8).

**Table 1:** AI procedures

Trial	Hormone delivery	Day 1	Day 3	Day 4	Pregnancy rate	Average litter size (range)	Survival rate
1	None	AI 5:00 pm			0 %	NA	NA
2	None	AI 8:00 pm			0 %	NA	NA
3	5 IU	PMSG 8:30 am	hCG 8:00 am AI 12:00 pm		0 %	NA	NA
4	5 IU	PMSG 8:30 am	hCG 8:00 am AI 5:00 pm		10 %	2.0 (2)	100 %
5	5 IU	PMSG 8:30 am	hCG 8:00 am AI 10:00 pm		0 %	NA	NA
6	5 IU	PMSG 12:00 pm	hCG 11:00 am AI 11:00 am		0 %	NA	NA
7	5 IU	PMSG 12:00 pm	hCG 11:00 am	AI 11:00 am	10 %	1.0 (1)	0 %
8	5 IU	PMSG 5:30 pm	hCG 5:00 pm	AI 9:00 am	30 %	10.0 (7–13)	100 %
9	5 IU	PMSG 8:30 pm	hCG 8:00 pm	AI 9:30 am	36 %	4.5 (2–9)	43 %
10	5 IU	PMSG 6:30 pm	hCG 6:00 pm AI 5:30 pm		0 %	NA	NA
11	1 IU	PMSG 8:30 am	hCG 8:00 am AI 5:00 pm		20 %	2.0 (2)	100 %
12	1 IU	PMSG 8:30 am	hCG 8:00 am AI 10:00 pm		40 %	5.0 (2–11)	100 %
13	1 IU	PMSG 8:30 pm	hCG 8:00 pm	AI 9:30 am	10 %	1.0 (1)	100 %
14	1 IU	PMSG 5:30 pm	hCG 5:00 pm	AI 9:00 am	50 %	5.0 (1–12)	100 %

Hormone concentrations, hormone administration, and sperm delivery schedules are shown for individual AI trials. Pregnancy rates, average litter size, the range of litter size, and the survival rate of pups to weaning age (21 days) are indicated. Sperm was delivered using the NSET device for mice without anesthesia. Analysis of data by Fisher’s exact test shows that a 13–16 h time interval between the hCG hormone injection and AI has a significant positive effect on pregnancy rates (*p* < 0.05) relative to other conditions tested. By the same test, administration of a hormone concentration of 1 IU has a significant positive effect on pup survival rate (*p* < 0.05) over 5 IU. No significant effect of hormone concentration on litter size or pregnancy rate is seen. Significance cannot be determined between individual trials due to insufficient sample size

With the aim of potentially increasing embryo implantation and improving the health of the pups resulting from the AI protocol, a decrease was made in the amount of hormones delivered to the female prior to sperm transfer. First, females in estrus were selected as AI recipients (N = 20). No hormones were administered to induce ovulation. AI was performed at 5:00 pm or at 8:00 pm (Table 1; Trials 1 and 2) before females were paired with vasectomized males overnight. No pups were obtained. Control matings with stud males confirmed that the females were receptive. The pregnancy rate for control matings was 50 % with an average litter size of 12 pups (N = 6). Next, female AI recipients were given low levels of hormones to induce a more natural ovulation condition; PMSG and hCG were reduced to 1 IU per i.p. injection, given 47 h apart. With the administration of a lower hormone dose, a trend was observed in improvement in pregnancy rate and pup health. The protocol with the highest pregnancy rate (Trial 14) followed a convenient time schedule for hormone injections and AI. 1 IU of PMSG was administered at 5:30 pm 3 days prior to sperm transfer and 1 IU of hCG was administered at 5:00 pm 1 day prior to sperm transfer to ovulate AI recipients. The next morning, sperm was collected at 8:00 am, capacitated for 1 h, and 40 µl delivered at 9:00 am transcervically to AI recipients. Recipients were immediately paired with vasectomized males overnight. Full term pregnancy rates with fresh sperm reached 50 % (N = 20) (Table 1; Trial 14). Litter size averaged 5.0 pups with all pups surviving to weaning.

## Discussion

A protocol refinement for nonsurgical AI in mice is described, which incorporates a convenient hormone administration schedule for female recipients and rapid, non-stressful sperm transfer without the need for anesthesia or analgesia. Sperm transfer was performed using the NSET device for mice, originally designed for nonsurgical transfer of embryos into the uterine horns of pseudopregnant female mice. The device consists of a flexible Teflon catheter that is able to pass the cervix and deposit embryos or sperm into the uterine horn. Utilization of the device for embryo transfer is considered a refinement (Steele et al. 2013) according to Russell and Burch’s ‘3Rs’ of animal research (Russell and Burch 1959) defining alternative procedures which either replace animals, reduce the number of animals used, or refine methodologies to minimize pain or distress. In the case of AI, use of the NSET device provides a convenient 3Rs refinement to a surgical procedure (Nakagata 1992; De Repentigny and Kothary 1996; Sato and Kimura 2001) and an alternative to a nonsurgical transcervical procedure using a blunt needle and glass speculum (Dziuk and Runner 1960), or a nonsurgical procedure requiring an artificial penis and vaginal tampon (Leckie et al. 1973; Takeshima and Toyoda 1977).

Nonsurgical AI is an assisted reproduction technique that has applications as a fertility treatment in humans and for commercial breeding of livestock. However, AI also has applications for use in laboratory mice. AI can be used for rapid expansion of mouse colonies as well as for generating age-matched cohorts for studies. Therefore, the use of AI may reduce the need for large in-house breeding colonies to provide mice for research projects. The ability to rapidly expand colonies on-site could reduce the cost of maintaining large breeding colonies. AI may be especially useful for breeding of transgenic or mutant mice with fertility problems and as an alternative to in vitro fertilization. Additionally, sperm can be shipped globally either cold (Takeo et al. 2012) or cryopreserved using one of two efficient protocols (Behringer et al. 2014), reducing the need to ship live animals. Nonsurgical AI could provide a means to rapidly recover a strain after shipment of sperm. Additionally, use of this nonsurgical AI protocol has potential to be coupled with thawed cryopreserved sperm. As reviewed in Takeo and Nakagata (2010), recent advances in cryopreservation of mouse strains have increased viability of thawed sperm. Therefore, transfer of thawed sperm with the NSET device has the potential to provide an alternative to IVF for strain cryorecovery.
